# Pheochromocytoma complicated with acute myocardial infarction and cerebral infarction: a case report

**DOI:** 10.3389/fonc.2026.1737241

**Published:** 2026-05-22

**Authors:** Yi Zhang, Fei Zhou, Hao Zhou, Jian Deng, Xiaoyu Chen, Xuemei Li, Rui Wang

**Affiliations:** 1 Department of Neurology, Affiliated Hospital of Shandong Second Medical University, Weifang, China; 2 Department of Emergency, Affiliated Hospital of Shandong Second Medical University, Weifang, China; 3 Electrocardiogram room, Affiliated Hospital of Shandong Second Medical University, Weifang, China

**Keywords:** case report, cerebral infarction, myocardial Infarction, myocardial infarction with non-obstructive coronary arteries, pheochromocytoma

## Abstract

**Background:**

Pheochromocytoma is a relatively rare neuroendocrine tumor originating from chromaffin cells of the adrenal medulla. Characterized by excessive catecholamine release, it typically presents with paroxysmal or sustained hypertension, palpitations, and other related symptoms. Catecholamine-mediated sustained hypertension can cause vascular endothelial injury, atherosclerotic plaque formation, and vasospasm, thereby increasing the risk of cardiovascular and cerebrovascular events. Cases of acute myocardial infarction and cerebral infarction at the same time are extremely rare. Owing to the lack of typical tumor-related symptoms, patients are easily misdiagnosed with primary hypertension complicated by cardiovascular and cerebrovascular diseases, leading to inappropriate clinical management.

**Case presentation:**

This case reports a 70-year-old female patient with acute ST-segment elevation myocardial infarction, who was initially treated with antiplatelet and anticoagulant therapy. The day after admission, she suffered from acute massive cerebral infarction and underwent emergency intracranial thrombectomy. During the operation, it was confirmed that the right internal carotid artery and superior trunk of the right middle cerebral artery were occluded, and a large amount of thrombus was removed. Further history taking revealed a prior diagnosis of right adrenal pheochromocytoma. Subsequent laboratory examination showed significant elevation of urinary vanillylmandelic acid. Coronary computed tomography angiography showed only mild stenosis, and the patient was considered to have myocardial infarction with non-obstructive coronary arteries and type 2 myocardial infarction. A subdural hematoma occurred after operation, and the patient’s condition gradually stabilized after dynamic adjustment of the antithrombotic regimen. The patient’s family refused surgical resection of the tumor. She had been taking phenoxybenzamine orally for a long time to control blood pressure after discharge. The patient’s prognosis was good during follow-up.

**Conclusions:**

Acute myocardial infarction and cerebral infarction were not independent events but rather sequential manifestations of systemic vasculopathy mediated by catecholamine storm. For patients with severe blood pressure fluctuations and unexplained multi-organ ischemia, screening for pheochromocytoma should be performed as soon as possible. Early identification, standardized use of α-blockers, and individualized adjustment of antithrombotic strategy are critical to improve the prognosis in such critically ill patients.

## 1 Introduction

Pheochromocytoma (PHEO) is a relatively rare neuroendocrine tumor originating from chromaffin cells in the adrenal medulla. Its core pathological feature is the paroxysmal or persistent release of large amounts of catecholamines, mainly including epinephrine and norepinephrine (1). According to epidemiological studies, the incidence of PHEO is approximately 1.9 per million person-years (2). It mainly occurs in individuals aged 30–50 years (3, 4), with a prevalence of about 0.2% – 0.6% in people with hypertension (5, 6). PHEO typically presents with persistent or paroxysmal hypertension, as well as the classic triad of headache, palpitations, and profuse sweating. Some patients may experience multiple organ dysfunction due to tumor compression or abnormal hormone secretion (7). Among the various complications of PHEO, cardiovascular and cerebrovascular damage has attracted significant attention due to its acute onset and poor prognosis. Excessive catecholamine secretion can induce a variety of atypical cardiovascular manifestations, leading to severe complications, such as ventricular tachycardia, acute myocardial infarction, heart failure, and stress cardiomyopathy. It can also trigger hypertensive crisis on the basis of catecholamine-mediated vasospasm or increased myocardial oxygen consumption (8–10). In addition, excessive catecholamines can also cause nervous system damage, such as cerebral infarction and cerebral hemorrhage, through cerebral vasospasm, violent fluctuation of blood pressure and hypercoagulable state (11–18). However, cases of PHEO-induced acute myocardial infarction combined with cerebral infarction are extremely rare. Due to the lack of typical tumor-related symptoms, it is easy to be misdiagnosed with primary hypertension complicated by cardiovascular and cerebrovascular diseases, which leads to inappropriate clinical management. This article reports a case of PHEO complicated with acute myocardial infarction and cerebral infarction, and describes its diagnosis, treatment process and key examination results in detail.

## 2 Case presentation

The patient was a 70-year-old female who was admitted to the hospital with the chief complaint of paroxysmal chest tightness and dyspnea for more than 10 years and exacerbation for 4 days. More than 10 years ago, the patient developed chest tightness and shortness of breath without any obvious triggers. After relevant examinations at a local hospital, the patient was diagnosed with coronary atherosclerotic heart disease. These symptoms recurred without special treatment. Four days before admission, the patient experienced recurrent chest tightness and breathlessness after getting cold. The symptoms persisted and did not relieve, accompanied by fever up to 40°C. After taking ibuprofen, her body temperature gradually decreased to normal, but she still had chest tightness, breathlessness, abdominal distension and general fatigue. The patient was admitted to the emergency department. Electrocardiogram (ECG) showed sinus tachycardia, pathological Q waves in leads V1 – V2, ST-segment elevation in a convex-upward pattern in leads V1 – V4, and T-wave inversion in leads V2 – V6 (**Figure 1A**). The patient was admitted to the hospital under the diagnosis of acute myocardial infarction. The patient had a history of type 2 diabetes for more than 10 years. She was treated with metformin hydrochloride 0.5 g twice daily and dapagliflozin 10 mg once daily, and her blood glucose level was well controlled. The patient also had a history of hypertension for 8 years, with a maximum blood pressure of 180/100 mmHg. She took antihypertensive drugs irregularly. However, she could not specify the drug names, and did not monitor blood pressure regularly. No smoking or alcohol addiction. There was no family history of similar diseases and genetic history.

**Figure 1:**
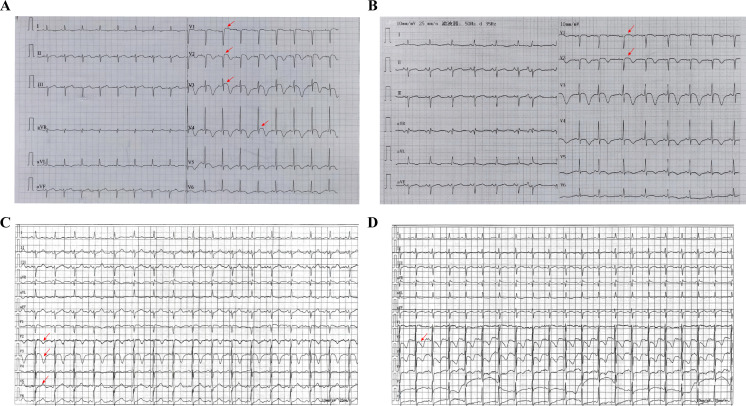
Electrocardiogram (ECG) evolution. **(A)** ECG in the emergency rescue room. Heart rate 102 beats per minute (bpm); sinus tachycardia; pathological Q waves in leads V1 and V2; ST-segments elevate in leads V1–V4; T-waves invert in leads V2–V6. **(B)** ECG on the second day of admission. Heart rate 95 bpm; sinus rhythm; occasional atrial premature contractions; pathological Q waves in leads V1 and V2; ST-segments elevate in leads V1–V4; T-waves invert in leads V2–V6; no significant changes compared to the admission ECG. **(C)** ECG on the third day of admission. Heart rate 93 bpm; sinus rhythm; left axis deviation; ST-segments fall in leads V1–V4 compared to previous ECG, with some segments transitioning from elevation to depression; T-waves invert in leads V3 and V4, gradually becoming upright in leads V5 and V6. **(D)** ECG on the fifth day of admission. Heart rate 110 bpm; sinus tachycardia; ST-segment depression in leads V2–V6; inverted T waves in leads V2 and V3; shallowly inverted or flattened T waves in leads V4–V6. **(A)**, the red arrows indicate ST-segment elevation in leads V1-V4. **(B)**, the red arrows denote arched upward ST-segment elevation accompanied by pathological Q waves in leads V1 and V2. **(C)**, the red arrows show that the ST-segments declined and T waves were inverted compared with prior examinations. **(D)**, the red arrows represent the decreased ST-segments. We appreciate your great patience and careful review.

Admission physical examination: temperature 36.0 °C, pulse 99 beats per minute, respiratory rate 18 beats per minute, blood pressure 115/61 mmHg, the body weight 44 kg. The patient was an elderly female, conscious, in poor general condition, and cooperative during the examination. No obvious abnormalities were found in the head, face, and five sense organs. The neck was soft without resistance. The breath sounds of both lungs were clear, and no obvious dry or moist rales were heard. The heart rate was 99 beats per minute, the rhythm was regular, and no pathological murmurs were heard in each valve area. The abdomen was soft, without tenderness or rebound, and bowel sounds were normal. Physical examination of the spine, limbs, and nervous system showed no obvious abnormalities. Preliminary diagnoses included coronary atherosclerotic heart disease, acute myocardial infarction, Killip class I, Grade 3 hypertension (very high risk) and type 2 diabetes mellitus.

The patient underwent relevant examinations on the day of admission, and the first blood test results (**Table 1**) were as follows: Blood routine examination: White blood cell count 8.47 × 10^9^/L, Neutrophil count 6.02 × 10^9^/L, Lymphocyte count 1.7 × 10^9^/L; Myocardial enzymes: Creatine kinase-MB 2.20 ng/mL, Myoglobin 118.20μg/L, High-sensitivity cardiac troponin T 57.90 ng/L; N-terminal pro-B-type natriuretic peptide: 16446.26 pg/mL; Procalcitonin (PCT): 20.000 ng/mL; Alanine aminotransferase 46.8 U/L, Aspartate aminotransferase 46.6 U/L; Serum sodium 136.0 mmol/L, Serum potassium 3.43 mmol/L. The result of routine urine and stool examinations were normal. Cardiac ultrasound showed an ejection fraction of 60%, with no significant segmental abnormalities in the left ventricular wall motion. On admission, the patient’s ECG was consistent with ST-segment elevation myocardial infarction (STEMI), but the chest tightness had persisted for 4 days, which was far beyond the optimal time window of emergency percutaneous coronary intervention. Additionally, her hemodynamics were stable without persistent ischemic symptoms. Therefore, the patients were treated with aspirin enteric-coated tablets 100 mg orally once daily combined with clopidogrel bisulfate tablets 75 mg orally once daily for antiplatelet therapy, and enoxaparin sodium injection 4000 IU subcutaneously once daily for anticoagulation therapy. At the same time, rosuvastatin calcium tablets 10 mg orally once daily for lipid regulation, nitrate drugs for vasodilation, hypoglycemic agents, rabeprazole sodium enteric-coated tablets to inhibit acid and protect gastric mucosa and other drugs were used for conservative treatment. The patient had a history of high fever after cold before admission, and the laboratory test results showed that PCT was significantly increased. But the physical examination and related auxiliary examinations did not find clear infection foci in the lungs, urinary system, digestive tract or other sites. Moreover, the patient’s body temperature did not rise again, and no abnormal inflammatory indicators were observed in the blood routine examination. Therefore, empirical anti-infection treatment was not given. After the above treatment, the patient’s chest tightness symptoms were improved.

**Table 1:** Laboratory test results.

Parameters		Patient values					Reference range
		Day 1	Day 4	Day 7	Day 14	Day 24	
Blood routine examination	WBC	8.47 × 10^9^/L	7.35 × 10^9^/L	8.08 × 10^9^/L	8.75 × 10^9^/L	8.04 × 10^9^/L	3.5–9.5 × 10^9^/L
	NEUT	6.02 × 10^9^/L	5.76 × 10^9^/L	5.98 × 10^9^/L	7.28 × 10^9^/L	5.76 × 10^9^/L	1.8–6.3 × 10^9^/L
	LYMPH	1.7 × 10^9^/L	1.5 × 10^9^/L	1.2 × 10^9^/L	1.2 × 10^9^/L	1.4 × 10^9^/L	1.1–3.2 × 10^9^/L
PCT		20.000 ng/mL	4.810 ng/mL	0.994 ng/mL	0.088 ng/mL	0.023 ng/mL	<0.05 ng/mL
NT-proBNP		16446.26 pg/mL	4575.12 pg/mL	3310 pg/mL	1087 pg/mL	540 pg/mL	0–125 pg/mL
ALT		46.8 U/L	74.4 U/L	69.9 U/L	31.4 U/L	27.3 U/L	7–40 U/L
AST		46.6 U/L	102.1 U/L	48.2 U/L	32.9 U/L	25.6 U/L	13–35 U/L
cTnT-hs		57.90 ng/L			16.70 ng/L		0–14 ng/L
CK-MB		2.20 ng/mL			6.60 ng/mL		0–5 ng/mL
MYO		118.20 μg/L			69.60 μg/L		0–70 μg/L
Na^+^		136.0 mmol/L	148.0 mmol/L	170.0 mmol/L	161.3 mmol/L	152 mmol/L	137–147 mmol/L
K^+^		3.43 mmol/L	3.25 mmol/L	2.63 mmol/L	3.50 mmol/L	4.0 mmol/L	3.5–5.3 mmol/L
Urinary VMA			59.11mg/24h				0.00–12.00mg/24h
Urinary CA			Negative				Negative

WBC, white blood cell; NEUT, neutrophil count; LYMPH, lymphocyte count; PCT, procalcitonin; NT-proBNP, N-Terminal Pro-Brain Natriuretic Peptide;, ALT, alanine aminotransferase; AST, aspartate aminotransferase; cTnT-hs, high-sensitivity cardiac troponin T; CK-MB, creatine kinase-MB; MYO, myoglobin; Na^+^, serum sodium; K^+^, serum potassium; VMA, Vanillylmandelic acid; CA, Catecholamine.

At 04:00 a.m. on the second day of admission, the patient suddenly developed choking while drinking water, slurred speech, and left limb motor dysfunction. Neurological examination showed drowsiness, dysarthria, left conjugate gaze palsy, shallow left nasolabial fold, deviated mouth, and tongue deviation to the left side. Muscle strength of the left upper limb grade 0, muscle strength of the left lower limb grade 1, sensory examination uncooperative, and positive bilateral pathological signs. Cranial magnetic resonance imaging showed a large-scale acute cerebral infarction in the right frontoparietotemporal insular lobe, as well as multiple stenoses and decreased branches of the right middle cerebral artery (**Figures 2A–C**). The patient was considered to have new-onset cerebral infarction, which was in accordance with the indications for interventional surgery. Cerebral angiography revealed occlusion of the right internal carotid artery and the superior trunk of the right middle cerebral artery (**Figures 2E, F**). During the operation, it was confirmed that the right upper trunk of the middle cerebral artery was occluded, and a large amount of thrombus was removed with an eTICI grade 3. After surgery, the patient was transferred to the Emergency Intensive Care Unit with an endotracheal intubation. To avoid the increased risk of intracranial hemorrhage caused by multiple antithrombotic drugs, aspirin, clopidogrel and enoxaparin sodium were discontinued after operation. Instead, tirofiban injection was administered via continuous intravenous infusion alone. Meanwhile, butylphthalide was used for cranial nerve nutrition, edaravone dexborneol was used for free radical scavenging, and fluid infusion was administered. The APACHE II score was 23. ECG (**Figures 1C, D**) was reexamined several times after surgery, showing that the ST-segment gradually fell from elevation to down, and the T wave changed from inverted to upright. On the third day after admission (the first day after operation), computed tomography was reexamined (**Figures 2D**, **3**). It revealed patchy low-density lesions in the right frontal lobe and insular lobe, indicating cerebral infarction. An ovoid soft tissue lesion was found between the liver and kidney, with poorly defined margins, measuring approximately 10 cm × 6.8 cm and containing irregular low-density areas. The right adrenal gland was not clearly identified. During this period, the patient’s blood pressure fluctuated greatly (**Figure 4A**), and the systolic blood pressure could reach up to 200 mmHg. Intermittent hypotension below 90/60 mmHg occurred, and the patient was treated to control blood pressure and dynamically adjust the infusion rate.

**Figure 2:**
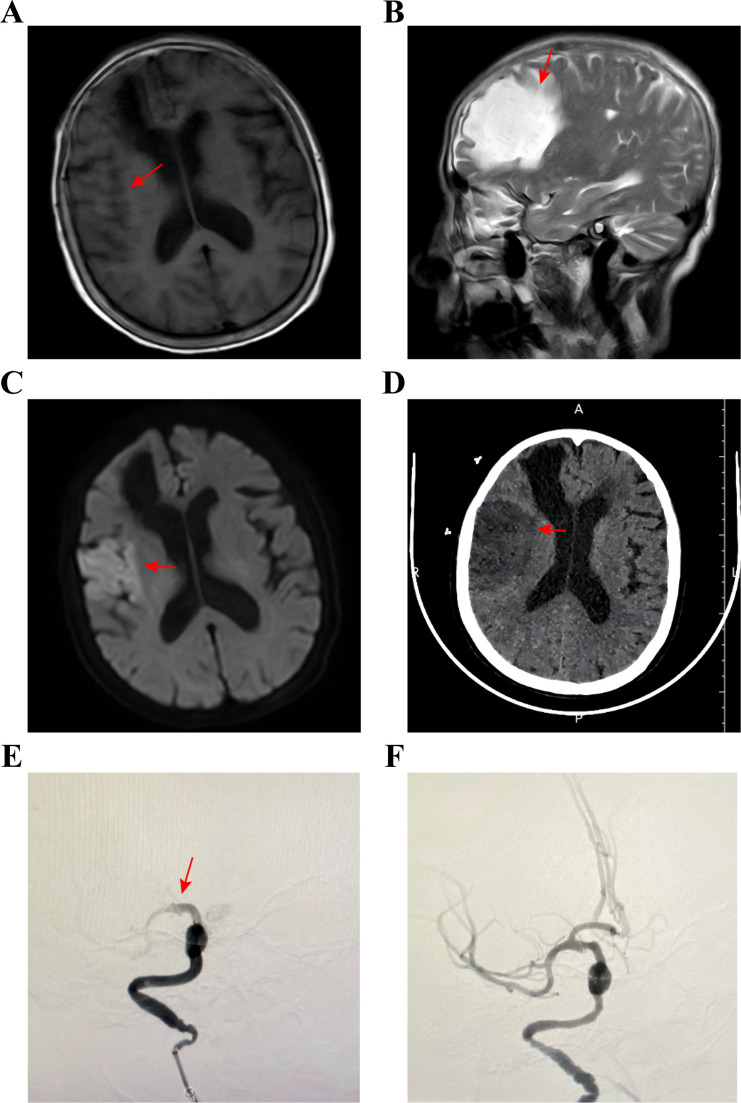
Findings of cranial magnetic resonance imaging, cranial computed tomography (CT), and percutaneous intracranial artery thrombectomy. **(A–C)** Cranial MRI findings (T1, T2, DWI sequences). Large-area acute cerebral infarction in the right frontal, parietal, temporal, and insular lobes; large-area slightly long T1 and slightly long T2 signals are seen in the right frontal, parietal, temporal, and insular lobes, with increased DWI signals; large-area long T1 and long T2 signals are seen in the right frontal lobe, with some slices communicating with the right lateral ventricle, and patchy long T1 and long T2 signals around the lesion. **(D)** Cranial CT findings: Patchy hypodense shadows are seen in the right frontal lobe and insular lobe, with blurred local cerebral sulcus and gyrus structure and gray-white matter junction, suggesting cerebral infarction changes. **(E, F)** Intraoperative findings of percutaneous intracranial artery thrombectomy. Occlusion of the superior trunk of the right middle cerebral artery; eTICI Grade 3 after thrombectomy.

**Figure 3:**
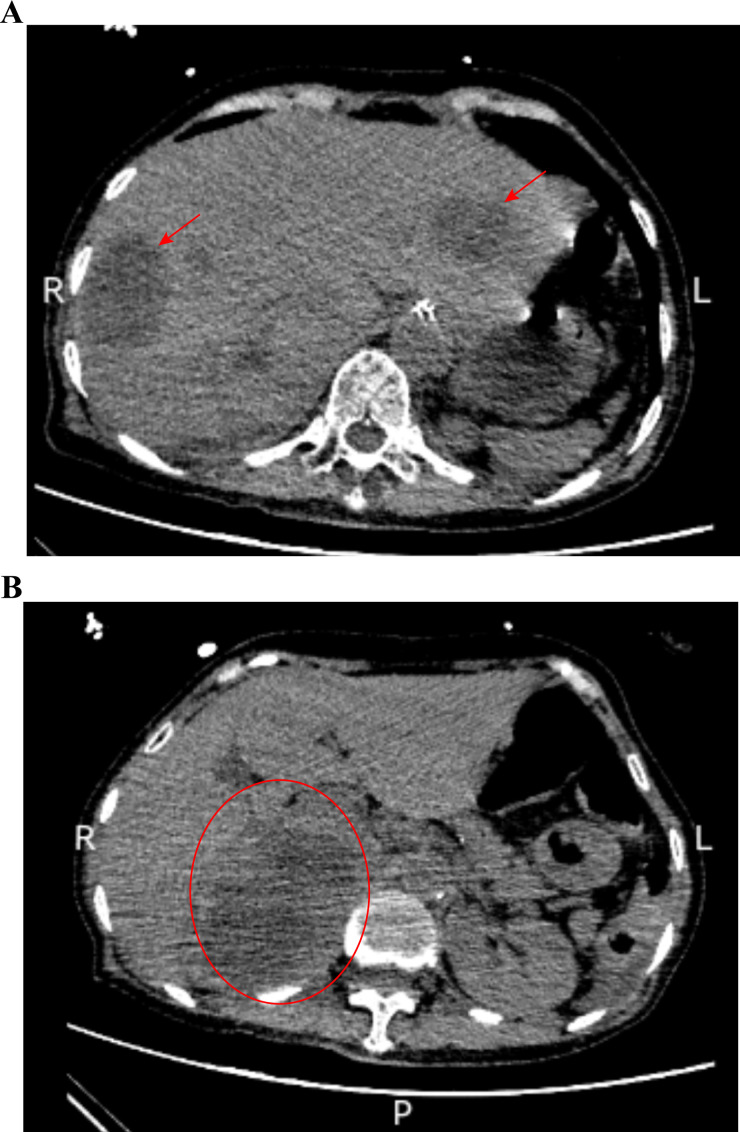
Findings of abdominal CT. **(A)** The size and shape of the liver are generally normal. Multiple low-density areas with ill-defined borders are present within the liver. **(B)** An oval-shaped soft tissue mass is visible between the liver and kidney. It contains irregular low-density areas. The right adrenal gland is not clearly visualized.

**Figure 4:**
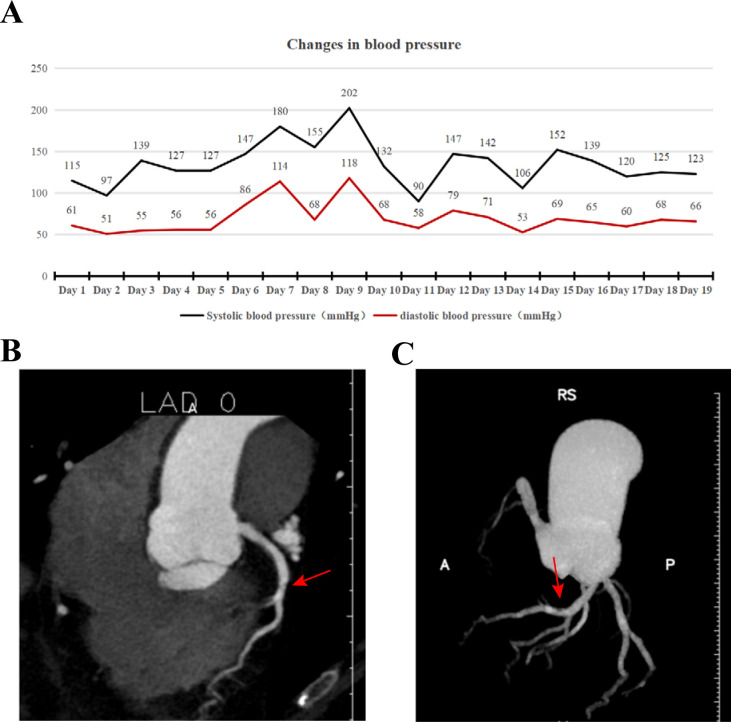
Blood pressure changes and findings of coronary CTA. **(A)** Blood pressure changes during hospitalization, with systolic pressure reaching a maximum of 202 mmHg (26.9 kPa). **(B, C)** Findings of coronary CTA: Mild stenosis in the proximal and mid segments of the left anterior descending artery; no plaque or stenosis observed in the distal segment.

The patient developed double cardiac and cerebral infarction within a short period. Further history obtained from the family revealed that the patient had been treated at Peking Union Medical College Hospital more than 2 months ago. PET/CT and relevant laboratory examination confirmed a diagnosis of right adrenal pheochromocytoma. Urinary vanillylmandelic acid (VMA) was subsequently measured: urinary VMA Content 59.11 mg/24h; Urinary catecholamine (CA): negative (**Table 1**). Sedative drugs were gradually stopped, the patient’s consciousness gradually improved, and spontaneous breathing restored. The endotracheal tube was removed on the fifth day of admission (the third day after operation). The patient remained afebrile postoperatively. Laboratory tests on the fourth day after the operation showed that PCT decreased significantly, cardiac function improved compared with before, blood routine examination did not show obvious abnormalities, and liver function parameters were mild elevated (**Table 1**). Cranial computed tomography revealed signs of a localized subdural hematoma along the right tentorium cerebelli, and tirofiban was stopped immediately. During hospitalization, the patient developed a central electrolyte disorder, with the serum sodium was up to 170 mmol/L. After nasal feeding with warm water and reducing sodium liquid, the serum sodium gradually decreased. After the patient’s condition was stable, further coronary computed tomography angiography (CTA) showed mild stenosis in the proximal and middle segments of the left anterior descending artery (**Figures 4B, C**). The patient was advised to undergo surgical treatment to deal with PHEO, but the family members refused after discussion, and she continued to receive conservative treatment with phenobenamine 20 mg orally twice daily. After conservative treatment, the cranial computed tomography reexamination 2 weeks after operation showed that the hematoma did not expand and was gradually resolving. Aspirin enteric-coated tablets 100 mg orally once daily was taken for antiplatelet aggregation treatment, and the patient was transferred to a general ward. Subsequent follow-up abdominal computed tomography showed that the PHEO lesion did not change significantly, and the size, morphology, and density of multiple low-density lesions in the liver did not change. Considering the patient’s critical condition, including catecholamine storm and cardiovascular and cerebrovascular infarction, the low-density lesions in the liver are most likely to be transient changes caused by acute stress. However, PHEO has the potential for liver metastasis, and the lesion did not change in the short term, so metastatic lesions cannot be completely excluded. On the twenty-fourth day of admission, the patient was clear and in stable general condition, with no chest tightness or dyspnea. Physical examination revealed slightly slurred speech and normal ocular movements. The pupils on both sides were of equal size and round with a diameter of approximately 3 mm. The direct and indirect light reflex was sensitive. The left nasolabial fold was slightly flattened, with the tongue protruded in the midline. The muscle strength of the left upper limb was grade 3, and that of the left lower limb was grade 4. Sensation was acceptable. No pathological reflexes were elicited bilaterally. The patient was subsequently discharged. After discharge, she took aspirin enteric-coated tablets 100 mg once daily for antiplatelet aggregation and phenobenzine 20 mg twice daily for blood pressure control, with regular follow-up. Telephone follow-up was conducted at 1, 6, and 12 months after discharge, and there were no serious adverse events.

## 3 Discussion

PHEO is a benign tumor derived from the neuroectoderm. The tumor contains chromaffin cells that produce and store catecholamines, which can be released either intermittently or continuously, causing paroxysmal or persistent hypertension and corresponding secondary clinical manifestations (18). Functional adrenal tumors, mainly PHEO, can secrete a mixture of epinephrine and norepinephrine or norepinephrine alone, and a few secrete dopamine (19, 20). Norepinephrine is often released relatively continuously, exerting its effects mainly through activation of α-adrenergic receptors in peripheral vascular. This often leads to sustained hypertension, and some may be associated with paroxysmal exacerbations (21, 22). In contrast, epinephrine secretion is influenced by multiple factors such as storage and release of tumor cells, and is often released in a pulsed fashion. It strongly stimulates cardiac β_1_ receptors and can induce tachyarrhythmia such as supraventricular tachycardia (23, 24). Life-threatening cardiovascular complications associated with PHEO are generally rare and atypical. Clinical manifestations may include angina pectoris, acute coronary syndrome changes on ECG, arrhythmias, elevated cardiac biomarkers and other myocardial infarction like changes. In severe cases, patients may develop transient left ventricular systolic dysfunction, heart failure, cardiogenic shock, as well as Takotsubo-like cardiomyopathy (25, 26). In addition, PHEO can also rarely present with aortic dissection (27) or be complicated by cerebrovascular events such as acute ischemic stroke (11–15, 28–30). Cases of acute myocardial infarction and cerebral infarction at the same time are extremely rare, and due to the lack of typical tumor-related symptoms, such patients are easily misdiagnosed with primary hypertension complicated by cardiovascular and cerebrovascular diseases, leading to inappropriate therapeutic strategies.

This case report describes a 70-year-old female patient with PHEO who presented with ST-segment elevation myocardial infarction outside the therapeutic time window, followed by sudden cerebral infarction within a short period (**Table 2**). Upon further inquiry of the medical history, the patient was diagnosed with right adrenal PHEO at another hospital two months prior ago. During this hospitalization, urinary VMA was significantly elevated. As a metabolite of catecholamines, the elevation of VMA confirms the persistent release of catecholamines by the tumor (31). Currently, the biochemical markers for the standard diagnosis of PHEO were plasma free metanephrine and normetanephrine as the first choice, whose sensitivity and specificity are better than the traditional urinary catecholamines and vanilmandelic acid (32). Based on the patient’s electrocardiographic findings, myocardial biomarkers, cardiac ultrasound, and coronary CTA results, the patient’s myocardial infarction was considered to be myocardial infarction with non-obstructive coronary arteries (MINOCA), which also corresponded to type 2 myocardial infarction (Type 2 MI). Her ECG showed ST-segment elevation with an upward convex configuration, and the high-sensitivity cardiac troponin T level was significantly elevated, which strongly suggested acute myocardial infarction. Subsequent coronary CTA showed only mild stenosis in the proximal and middle segments of the left anterior descending artery, without more than 50% obstructive lesions, which met the diagnostic criteria of MINOCA. The patient experienced catecholamine storm from PHEO, infectious stress, tachycardia, and blood pressure fluctuations. The combined effect of these factors resulted in an imbalance between myocardial oxygen supply and consumption, consistent with the core mechanism of type 2 MI. Unfortunately, the patient and her family refused to undergo subsequent coronary angiography and intracavitary imaging examinations, which could not absolutely rule out plaque rupture or intracoronary microthrombosis. However, the current evidence did not support type 1 myocardial infarction. Previous reports have mentioned that PHEO can cause MINOCA (33–39). Among them, Ku et al. (33), Melson et al. (38), and Karatzia et al. (40) reported that PHEO may initially present as MINOCA in the absence of significant coronary stenosis. The clinical manifestations were chest pain or chest tightness and T-wave changes in ECG, which were highly consistent with the presentation of this case. The mechanism of myocardial infarction is considered to be related to the excessive release of catecholamine. Excess catecholamines can activate α_1_-receptors on coronary vascular smooth muscle, which can lead to intense vasoconstriction and coronary vasospasm (41), and long-term vasospasm reduces coronary flow reserve. Catecholamines activate myocardial β_1_-receptors, leading to increased heart rate and myocardial contractility (41). This dramatically increases myocardial oxygen consumption, which contrasts with reduced coronary blood supply and exacerbates myocardial cell necrosis. In addition, some studies have found that sustained hypertension caused by catecholamines can directly damage coronary endothelial cells, destroy endothelial barrier function, accelerate the instability of atherosclerotic plaques, and reduce the secretion of endothelial anticoagulant substances. This promotes the adhesion and activation of platelets to form local thrombi, which further blocks the coronary artery (42).

**Table 2:** Timeline of diagnosis and management.

Time point	Event	Key manifestations/interventions
4 days before admission	Symptom Onset	Chest distress, dyspnea, and fever.
Day 1	STEMI	Intervention: Antithrombotic therapy initiated. Aspirin 100 mg qd, clopidogrel hydrogen sulfate 75 mg qd, and enoxaparin sodium 4000 IU qd subcutaneously.
Day 2 (4:00 a.m.)	Cerebral Infarction	Cerebral angiography revealed occlusion of the right internal carotid artery and superior trunk of the right middle cerebral artery. Intervention: Emergency mechanical thrombectomy. Oral antiplatelet and anticoagulant agents were discontinued postoperatively, with only intravenous tirofiban infusion.
Day 3	Pheochromocytoma	Abdominal CT showed a right adrenal mass (approximately 10 × 6.8 cm). Urinary vanillylmandelic acid was 59.11 mg/24 h.
Day 5	Extubation	
Day 6	Subdural Heatoma	Cranial CT showed a localized subdural hematoma along the right tentorium cerebelli. Intervention: Tirofiban was discontinued.
Day 16	Antithrombotic Resumption	The hematoma remained stable without expansion and showed gradual resorption. Single−antiplatelet therapy with aspirin 100 mg qd was restarted. Transferred to general ward.
Day 24	Discharge	Muscle strength: grade 3 in the left upper extremity and grade 4 in the left lower extremity. Intervention: Discharge medications, aspirin 100 mg qd for antiplatelet aggregation, phenoxybenzamine 20 mg twice daily for blood pressure control.
1, 6, and 12 months after discharge	Follow-up	No severe adverse events.

STEMI, ST-segment elevation myocardial infarction; CT, Computed Tomography.

The new-onset cerebral infarction was considered to be the result of the interaction of multiple factors. The patient had a long history of type 2 diabetes mellitus and hypertension, which are the major risk factors for intracranial atherosclerosis and cerebrovascular events. Suspected infection before admission leads to stress and a hypercoagulable state, which, in the context of massive catecholamines released by PHEO, causes cerebral vasospasm, blood pressure fluctuation, cerebral hypoperfusion, and ultimately induces acute cerebral infarction (18). Although the fever had resolved at admission, as the initial stress event, the preceding infection may have triggered the fulminant release of catecholamine and precipitated acute myocardial infarction. PCT was significantly increased on admission, but decreased rapidly in the absence of antimicrobial therapy, infection foci, fever, and elevated peripheral inflammatory markers. This suggests that the increase in PCT was more closely associated with catecholamine storm and sterile inflammation related to acute myocardial infarction, with cerebral infarction occurring as a subsequent event. During stress, the body’s coagulation system is activated, platelet aggregation is enhanced, and hypercoagulable state is promoted (43). At the same time, vasospasm and endothelial injury are aggravated, and microthrombus fusion is accelerated, which eventually leads to occlusion of the upper trunk of the middle cerebral artery. Resolution of the occlusion with eTICI grade 3 reperfusion following thrombectomy also confirmed that thromboembolic occlusion represented the direct pathological lesion. The TOAST classification of cerebral infarction in this case was most consistent with other determined etiology. Although the patient had underlying intracranial atherosclerosis, the core trigger for infarction was catecholamine storm secondary to PHEO. This led to a systemic hypercoagulable state, cerebral vasospasm, and violent blood pressure fluctuations, which precipitated acute thrombosis and embolism on the basis of preexisting stenosis (18). In the pathogenesis, arterial thromboembolism was the direct cause, cerebral vasospasm was the key aggravating factor, and cerebral hypoperfusion was the synergistic factor. Due to the absence of vessel wall imaging, rupture of large artery atherosclerotic plaque cannot be completely ruled out, but the comprehensive evaluation is that PHEO-related causes were mainly. Previous reports have indicated that PHEO can be complicated with acute cerebrovascular disease and systemic arterial thrombosis, and the mechanisms are closely related to excessive catecholamines, vasospasm, and hypercoagulable state (18, 23).

In this patient, myocardial infarction and cerebral infarction occurred within a short period. These were not isolated events, but rather distinct organ manifestations of systemic vascular disease caused by catecholamine excess due to PHEO, which also illustrates the vicious cycle between myocardial infarction and cerebral infarction (44) (**Figure 5**). Excessive catecholamines can induce systemic vasospasm, vascular endothelial injury, hypercoagulable state and violent fluctuations in hemodynamics, constituting a unified pathophysiological pathway. The only difference is that the involved vessels are coronary arteries and cerebral arteries. Catecholamines mediate cerebral vasospasm through α_1_-receptors, which may lead to vascular remodeling, luminal stenosis, and decreased cerebral blood flow reserve, and promote microthrombosis on the basis of endothelial injury, which may be a hidden risk for subsequent large thrombus occlusion (16, 18). Myocardial infarction itself impaired left ventricular systolic function and reduced cardiac output, which reduced the blood supply to the brain. In addition, fluctuating blood pressure during hospitalization exacerbated vasospasm during the high-pressure period, followed by insufficient cerebral perfusion during the low-pressure period. It finally led to the complete interruption of blood flow in the middle cerebral artery territory and catalyzed the occurrence of acute ischemic stroke (45). The stress response elicited by cerebral infarction in turn exerts adverse effects on the heart, exacerbating myocardial injury and blood pressure fluctuations, thereby establishing a vicious cycle of heart and brain injuries. This heart-brain interaction mechanism based on PHEO has rarely been systematically described in previous case reports. Regarding etiological differentiation, acute myocardial infarction was primarily attributed to catecholamine-induced coronary vasospasm, characterized by elevated myocardial injury markers, absence of significant obstructive lesions on coronary angiography or coronary CTA, and frequent reversibility following tumor resection and sympathetic blockade (18, 24, 26, 46). Although plaque rupture cannot be completely excluded, there was no thrombotic evidence. In the nervous system, multiple intracranial arterial stenoses and removal of abundant thrombus during operation indicate that arterial thromboembolism is the direct mechanism of cerebral infarction, and catecholamine-mediated cerebral vascular stenosis, hypercoagulable state, and cerebral hypoperfusion after myocardial infarction play a synergistic role.

**Figure 5:**
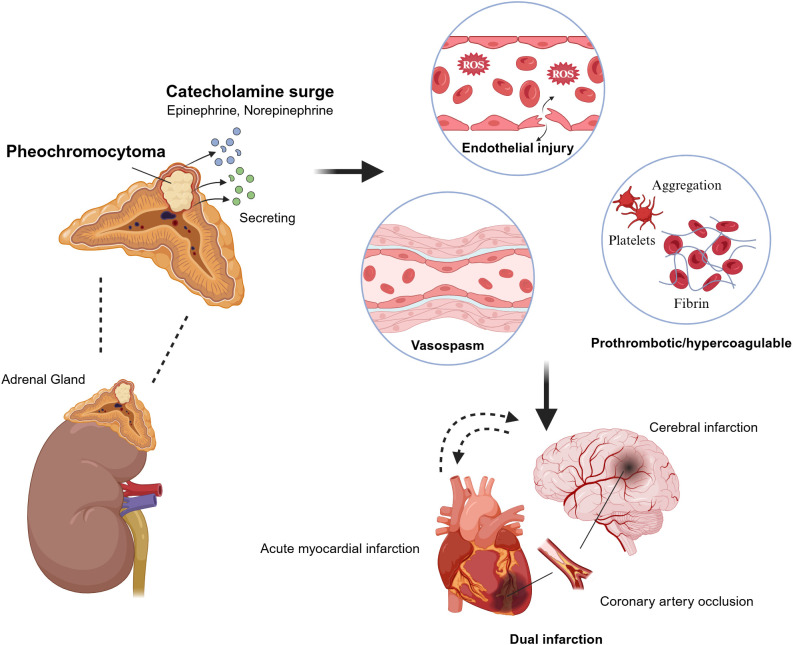
Schematic diagram of the mechanism of pheochromocytoma-induced cardiac and cerebral ischemia. Created in BioRender. Zhang, Y. (2026). https://BioRender.com/anri4di.

Treatment for excessive catecholamine secretion is divided into acute phase and long-term management. In the acute phase, α-adrenergic blockers are the first-line agents for blood pressure control, including phenoxybenzamine and phentolamine (47). β-blockers can be added if arrhythmias are present, but only in addition to α-adrenergic blockers to prevent life-threatening hypertensive crisis resulting from unopposed β-blockade alone (7). Long-term treatment is mainly based on radical surgical resection. For those who cannot be operated, long-term α-adrenergic blockers is indicated, and malignant cases can be combined with targeted or radionuclide therapy (7, 48). After the diagnosis of PHEO, the patient began to take phenoxybenzamine 10 mg twice daily to reduce blood pressure, and then maintained at a dosage of 20 mg twice daily. In the early stage of the disease, priority was given to stabilizing cardiovascular and cerebrovascular emergencies and vital signs. When the condition was stable, the patient and her family refused surgery temporarily. For patients who are not suitable for surgery, long-term standardized use of α-adrenergic blockers, along with monitoring of catecholamine levels and heart-brain function, is necessary to reduce the risk of target organ damage. Early diagnosis and prompt surgical resection of PHEO are the fundamental measures to prevent cardiovascular and cerebrovascular complications. Medical therapy constitutes the cornerstone of long-term management, which can mitigate persistent catecholamine-induced cardiovascular and cerebrovascular injury and prevent the recurrence of infarction. Following discharge, the patient continued phenoxybenzamine for blood pressure control, and the follow-up prognosis was good.

When PHEO complicated with acute myocardial infarction and cerebral infarction, antithrombotic therapy needs to weigh the risk of thrombosis, bleeding risk and surgical candidacy, resulting in a highly individualized treatment strategy. In patients presenting with MINOCA, the underlying mechanisms of infarction are primarily vasospasm, endothelial injury, and hypercoagulable state, which differ from those of typical type 1 myocardial infarction caused by plaque rupture. Consequently, antithrombotic regimens should not be completely equivalent to conventional acute coronary syndrome (36, 49). In this case, antithrombotic therapy was performed in four stages (**Table 2**). In the early stage, because the onset time had exceeded the optimal time for primary percutaneous coronary intervention, conservative medical treatment was adopted, including dual antiplatelet therapy and enoxaparin anticoagulation. Given the patient’s low body weight, a single dose of enoxaparin 40 mg once daily was close to the standard treatment dose, but the frequency of dosing was halved to reduce the cumulative risk of bleeding while addressing the hypercoagulable state (50). Emergency thrombectomy was performed after new cerebral infarction without stent implantation. To prevent intracranial hemorrhage, only short-term monotherapy with tirofiban was administered. Upon the development of subdural hematoma, all antithrombotic drugs were suspended immediately. After resolution of the hematoma and clinical stabilization, only aspirin monotherapy was resumed for secondary stroke prevention. In previous studies, there was no unified antithrombotic standard for PHEO with multiple thrombotic events, and most of them used individualized regimens (51, 52). This case suggests that when PHEO complicated with multi-organ ischemic events, antithrombotic therapy should focus on etiological treatment, dynamic evaluation of bleeding risk and timely adjustment of medication to improve prognosis. In underweight patients, doses should be calculated precisely, with appropriate dose reduction or simplified regimens based on the risk profile.

## 4 Strengths and limitations

This case report describes the diagnosis and treatment process of a patient with PHEO complicated by cardiac and cerebral infarction. With complete and thorough clinical data, it illustrates four successive dynamic adjustments to the antithrombotic regimen in accordance with clinical progression, providing an operable practical reference for the treatment of such complex cardiovascular and cerebrovascular complications. At the same time, this case systematically illustrates the vicious cycle mechanism of heart-brain interaction related to PHEO, which has rarely been systematically described in previous literature. However, these results are based on a single patient and have limited extrapolation. Furthermore, the patient and family declined coronary angiography, and coronary CTA has limited accuracy in evaluating plaque morphology in mild stenotic lesions. As a result, type 1 myocardial infarction cannot be completely excluded, which represents another limitation of this study. In addition, biochemical validation was not sufficient to complete plasma free metanephrine and normetanephrine testing. The patient’s family refused surgical treatment, so the long-term prognosis of cardiovascular and cerebrovascular events after tumor resection could not be evaluated.

## 5 Conclusions

In this case, the patient with PHEO developed acute myocardial infarction and cerebral infarction, mainly due to the continuous release of catecholamines from the tumor, which led to vasospasm, endothelial injury, and hypercoagulable state. Hemodynamic fluctuations and stress subsequent to myocardial infarction further precipitated simultaneous infarction in both cardiac and cerebral organs. In clinical practice, PHEO should be suspected in patients with severe blood pressure fluctuation and unexplained multi-organ ischemia. Plasma free metanephrine assays and abdominal imaging should be performed as soon as possible. Early diagnosis, administration of α-adrenergic blockers to stabilize blood pressure, and elective tumor resection are the keys to prevent recurrence of cardiovascular and cerebrovascular events. During treatment, antithrombotic strategies should be highly individualized and dynamically adjusted according to the clinical course, balancing thrombosis prevention and bleeding risk control. Once PHEO is diagnosed, it is important to closely track catecholamine levels along with cardiac and cerebral function. New-onset chest pain or neurological symptoms should raise suspicion of target organ damage.

## 6 Patient perspective

On the second day after percutaneous intracranial thrombectomy, the patient’s consciousness gradually recovered and she gave feedback on treatment. She said, “My left limb was completely paralyzed at the onset of cerebral infarction at night. Now I can move my fingers slowly, but I still can not lift them off the bed.” On the day of discharge, the patient said, “All my previous discomfort has gone away, and I can lift my left limb off the bed much better than before.” During a telephone follow-up 6 months after discharge, the patient said, “I still have slight weakness on my left side, but my quality of life has not been affected. At present, the blood pressure is controlled well, and surgery will be considered in the future.”

## Data Availability

The original contributions presented in the study are included in the article/**Supplementary Material**. Further inquiries can be directed to the corresponding authors.
